# Paraquat Modulates Alternative Pre-mRNA Splicing by Modifying the Intracellular Distribution of SRPK2

**DOI:** 10.1371/journal.pone.0061980

**Published:** 2013-04-16

**Authors:** Silvia Vivarelli, Silvia C. Lenzken, Marc-David Ruepp, Francesco Ranzini, Andrea Maffioletti, Reinaldo Alvarez, Oliver Mühlemann, Silvia M. L. Barabino

**Affiliations:** 1 Dipartimento di Biotecnologie e Bioscienze, Università di Milano – Bicocca, Milano, Italy; 2 Department of Chemistry and Biochemistry, University of Bern, Bern, Switzerland; International Centre for Genetic Engineering and Biotechnology, Italy

## Abstract

Paraquat (PQ) is a neurotoxic herbicide that induces superoxide formation. Although it is known that its toxic properties are linked to ROS production, the cellular response to PQ is still poorly understood. We reported previously that treatment with PQ induced genome-wide changes in pre-mRNA splicing. Here, we investigated the molecular mechanism underlying PQ-induced pre-mRNA splicing alterations. We show that PQ treatment leads to the phosphorylation and nuclear accumulation of SRPK2, a member of the family of serine/arginine (SR) protein-specific kinases. Concomitantly, we observed increased phosphorylation of SR proteins. Site-specific mutagenesis identified a single serine residue that is necessary and sufficient for nuclear localization of SRPK2. Transfection of a phosphomimetic mutant modified splice site selection of the E1A minigene splicing reporter similar to PQ-treatment. Finally, we found that PQ induces DNA damage and *vice versa* that genotoxic treatments are also able to promote SRPK2 phosphorylation and nuclear localization. Consistent with these observations, treatment with PQ, cisplatin or γ-radiation promote changes in the splicing pattern of genes involved in DNA repair, cell cycle control, and apoptosis. Altogether, our findings reveal a novel regulatory mechanism that connects PQ to the DNA damage response and to the modulation of alternative splicing via SRPK2 phosphorylation.

## Introduction

Parkinson's Disease (PD) is the second most common progressive neurodegenerative disorder of the central nervous system. Epidemiological studies suggest that PD is a multifactorial disorder probably arising from polygenic inheritance and gene–environmental interactions. Exposure to pesticides and to the herbicide paraquat (PQ, 1,1′-dimethyl-4,4′-bipyridinium) is known to increase the risk of developing PD. PQ uncouples the mitochondrial electron transport chain, which induces superoxide formation [1]. Thus its toxic properties support the hypothesis that neuronal damage in PD may arise from a mechanism of oxidative stress. Since in recent years PQ has become an increasingly popular model for studying the etiology of PD (15, 16), it is important to understand the molecular mechanism underlying PQ-induced toxicity to neural cells.

Recently, we reported that treatment of the human neuroblastoma cells SH-SY5Y with PQ induces extensive changes in alternative pre-mRNA splicing (AS) [2]. Pre-mRNA splicing is a crucial step of eukaryotic gene expression that has emerged in recent years as a major regulatory mechanism of cell cycle and apoptosis [3], [4]. Changes in AS have been observed in PD and in other neurodegenerative disorders [5]. Indeed, in response to cellular stress AS can be controlled by specific signal transduction pathways that lead to post-translational modifications of splicing factors and to changes in their activity and/or subcellular localization [6].

SR protein kinases (SRPKs) are a family of protein kinases that phosphorylate serine-arginine-rich proteins (SR proteins), which are important regulators of alternative splicing [7]. While SRPK1 is predominantly expressed in pancreas, SRPK2 is highly expressed in brain, and both are co-expressed in other human tissues and in many experimentally used cell lines [8]. SRPK1 and 2 are predominately localized in the cytoplasm, where they phosphorylate SR proteins that can thus be re-imported into the nucleus [9]. SRPK1 and 2 are highly similar proteins that contain a bipartite kinase domain separated by a unique spacer region. It has been shown that removal of the spacer in SRPK1 has little effect on the kinase activity, but triggers the translocation of the protein to the nucleus and consequently induces aggregation of hyperphosphorylated SR proteins in nuclear speckles [10]. Nuclear translocation of SRPK1 was recently reported to occur also upon Akt activation by EGF treatment [11].

Here we show that PQ treatment of SH-SY5Y human neuroblastoma cells leads to the re-localization of SRPK2 to the cell nucleus, to SR protein phosphorylation and to their accumulation in nuclear speckles. We find that phosphorylation of a specific serine residue is necessary and sufficient to localize SRPK2 to the nucleus and to modify the alternative splicing pattern of a minigene splicing reporter. In addition, we show that PQ treatment induces formation of H2AX foci that are indicative of DNA double-strand breaks. Consistent with this, we observe that also cisplatin and gamma irradiation induce nuclear accumulation of SRPK2. Collectively, these data indicate that PQ-induced AS changes are mediated by modificaton and relocalization of SRPK2 that phosphorylate splicing factors.

## Results

### Treatment with PQ induces modifications in the intracellular distribution and in the phosphorylation status of splicing factors

**Figure 1:**
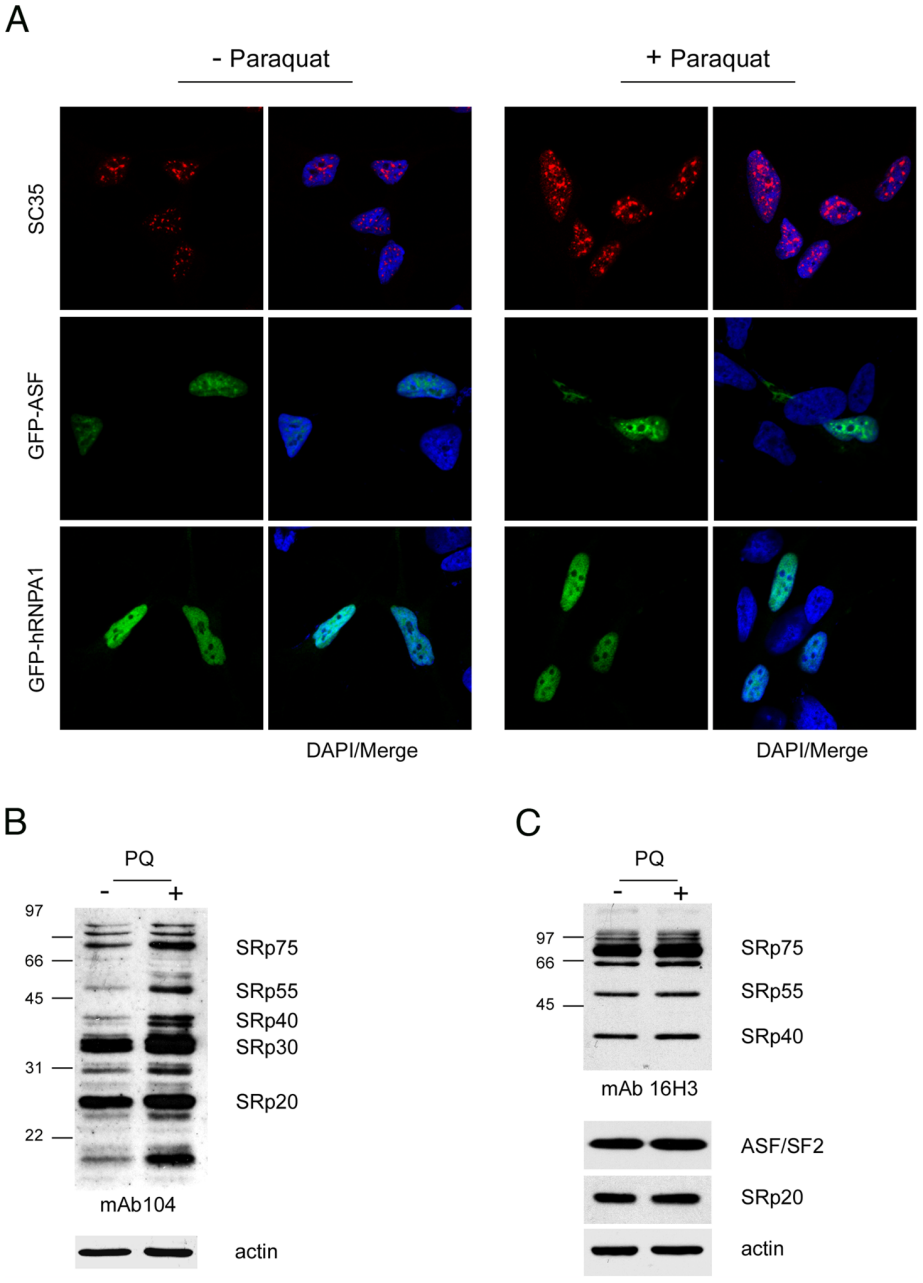
Increased phosphorylation of SR proteins in PQ-treated cells. A. PQ induced relocalization of SR proteins in the nucleus of treated cells. SH-SY5Y cells treated with vehicle or with 0.75 mM PQ for 18 h were immunostained with an anti-SC35 antibody (*upper row*), transiently transfected with GFP-ASF/SF2 (*middle row*), or with GFP-hnRNPA1 (*lower row*). Nuclei were stained with DAPI. B. Increased phosphorylation of SR proteins in PQ-treated cells. Total extract of control or PQ-treated cells was probed with mAb104 to determine the phosphorylation state of classical SR proteins. C. The same extracts used for the Western blot shown in panel B were probed with the 16H3 monoclonal antibody that detects SR proteins regardless of their phosphorylation status, with anti ASF/SF2 and anti-SRp20 monoclonal antibodies. Actin was used as loading control.

In a recent report we described the result of a splicing-sensitive microarray analysis of human neuroblastoma SH-SY5Y cells treated with paraquat (PQ) [2]. This analysis, despite detecting extensive changes in alternative splicing, did not show differential expression of any gene encoding splicing regulatory factors. This finding prompted us to test whether PQ induced posttranslational modifications and/or changes in the intracellular distribution of specific splicing regulators. We examined by immunofluorescence microscopy the intracellular distribution of several splicing regulatory proteins in cells incubated with 0.75 mM PQ for 18 hours. Using a monoclonal antibody that specifically recognizes phosphorylated SC35, we detected enlarged nuclear speckles (Figure 1A, upper row, and Figure S1). Relocalization of SR proteins to nuclear speckles in PQ-treated cells was further confirmed by the analysis of the distribution of GFP-ASF/SF2 in treated cells (Figure 1A, middle row). In contrast, PQ did not affect the intracellular distribution of members of the hnRNP family of splicing regulators (such as hnRNP A1, Figure 1A, bottom row, and hnRNP H and hnRNPK, data not shown), which has been reported to relocate to the cytoplasm following diverse types of stress treatments [12], [13]. We also checked expression of different hnRNP proteins by western blotting without detecting any significant variation (Figure S2).

Formation of enlarged nuclear speckles has been previously linked to hyperphosphorylation of SR proteins [14], [15]. We thus tested the phosphorylation status of SR proteins by western blotting using mAb104, a monoclonal antibody that specifically recognizes the common phosphoepitopes of classical SR proteins [16]. In response to PQ treatment, we observed an increase in the signal for all the classical SR proteins recognized by the antibody (Figure 1B). To check for possible alterations of protein levels upon PQ treatment, SR proteins were also visualized with the 16H3 antibody, which recognizes RS domains of different SR proteins regardless of their phosphorylation status, and with anti–ASF/SF2 and anti-SRp20 antibodies (Figure 1C). Since the protein levels of the SR proteins remained unchanged, our results collectively demonstrate that PQ treatment increases the phosphorylation of SR proteins.

**Figure 2:**
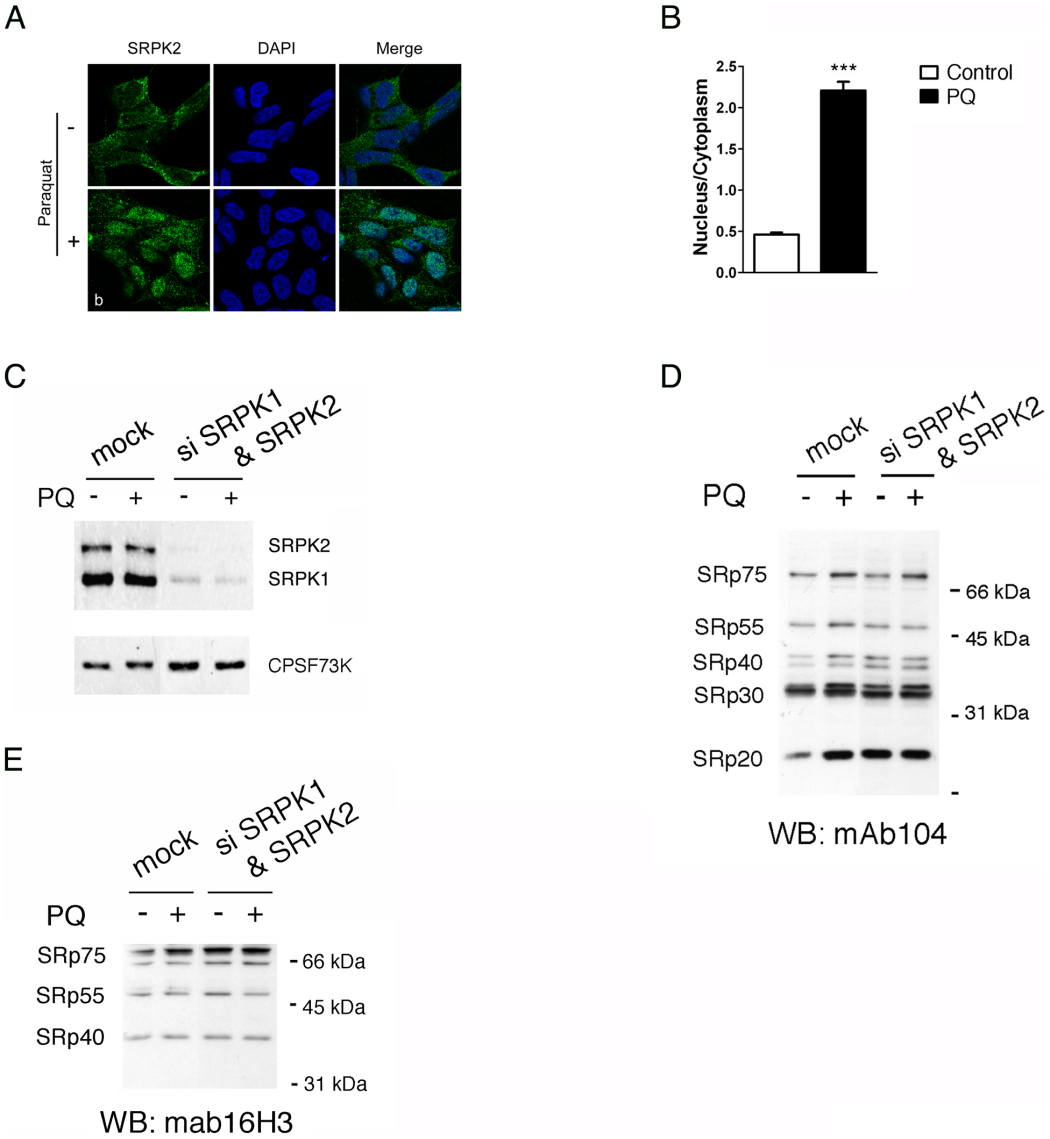
Nuclear translocation of SRPK2 and phosphorylation of SR proteins in response to PQ. A. Representative confocal micrographs of SH-SY5Y cells stained with an SRPK2-specific antibody. *Upper row*: cells treated with vehicle; *lower row*: cells treated with 0.75 mM PQ for 18 h. DAPI was used to identify the nuclei. B. The average nuclear to cytoplasmic ratio (N/C) ratio of SRPK2 fluorescent signal was determined for 50 cells as described in Materials and Methods. *** indicates p<0.001 treated vs. control group by unpaired t-test. C. RNA-mediated silencing strongly reduced SRPK1 and SRPK2 expression. Western blot analysis of the expression level of SRPK proteins in the same extract used for the Western blot shown in figure 2D and 2E. CPSF73K was used as loading control. D. Silencing of SRPK1 and SRPK2 abolishes PQ-mediated phosphorylation of SR proteins. Western blot analysis of nuclear extracts prepared from control SH-SY5Y cells treated with vehicle or with PQ, and from cells depleted of both SRPK1 and SRPK2. Phosphorylated SR proteins were detected with mab104. E. To control for equal loading of the samples SR proteins were also detected with mab16H3.

To date, several kinases have been reported to phosphorylate SR proteins. These include DNA topoisomerase I [17], SRPK1–3 [8], [18]–[20] and the family of CLK1/Sty kinases [21]. Upon PQ treatment we did not detect any appreciable change in either the expression or the intracellular distribution of CLK1/Sty (data not shown). In contrast, PQ induced the accumulation of SRPK2 in the cell nucleus (Figure 2A). Quantification of fluorescence images from individual cells revealed that the nuclear to cytoplasmic (N/C) ratio of the SRPK2 signal was ∼0.4 in untreated cells and ∼2.3 in cells treated with PQ (Figure 2B). To test if the observed increase in SR protein phosphorylation was due to SRPK activity, we knocked down both SRPK1 and SRPK2 using specific shRNAs (Figure 2C). Silencing was more efficient for SRPK2 than for SRPK1. We then used the phospho-specific antibody mAb104 to test the effect of PQ on the phosphorylation of SR proteins in SRPK depleted cells. As shown in Figure 2D, PQ treatment no longer resulted in increased phosphorylation of SRp55, SRp40, SRp30 and SRP20 in cells depleted of the two kinases. Interestingly, PQ was still able to promote phosphorylation of SRp75, suggesting that SRPKs are not redundant and have some substrate specificity. To control for equal loading of the samples, SR proteins were also visualized with the 16H3 antibody, which recognizes SR proteins regardless of their phosphorylation status (Figure 2E).

### SRPK2 phosphorylation at the Ser-581 residue is required for its translocation to the nucleus after paraquat treatment

**Figure 3:**
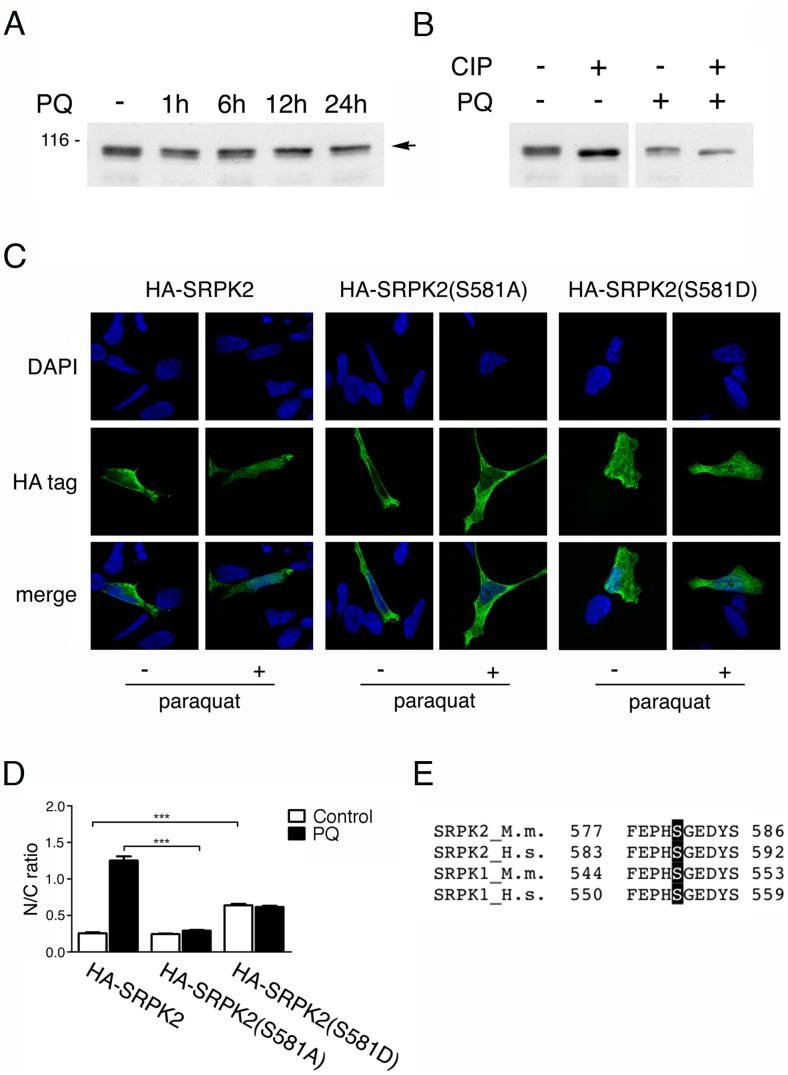
Phosphorylation of Ser581 is required for the nuclear localization of SRPK2. A. Western blots of total cell extract prepared from untreated SH-SY5Y cells or from cells treated with PQ for the indicated times. The blot was probed with an anti-SRPK2 antibody. Upon PQ treatment an increase of the slower migrating band (indicated by an arrowhead) could be observed. B. SRPK2 phosphorylation was confirmed by CIP treatment. Total cell lysates prepared from untreated cells and from cells treated with PQ were incubated with calf intestinal phosphatase (CIP) as described in Material and methods. C. Representative confocal micrographs of SH-SY5Y cells transfected with a construct expressing HA-tagged SRPK2, HA-SRPK2(S581A), or HA-SRPK2(S581D). *Upper row*: DAPI; *middle row*: cells stained with an HA-specific antibody; *lower row*: merge of the DAPI and antibody signals. D. The nuclear to cytoplasmic ratio (N/C) of the fluorescence signal was determined for 50 transfected cells. The graph shows the average N/C ratio of SRPK2 signal measured as described in Materials and Methods. E. *** indicates p<0.0001 of groups compared by one-way ANOVA and Tukey post-test analysis. F. Sequence alignment of the 577–586 aa region in SRPK2 highlighting conservation of CK2 consensus sites across species. A black, vertical bar indicates the conserved serine.

SRPK2 normally appears on SDS-PAGE as two closely migrating bands. In extracts prepared from PQ-treated cells we observed an increased intensity of the slower migrating SRPK2 species at the expense of the faster migrating species (Figure 3A). To determine whether the mobility shift of SRPK2 was due to increased phosphorylation, the extracts were treated with calf intestinal phosphatase (CIP). After incubation with the phosphatase, the slower migrating SRPK2 band in both the untreated and the PQ-treated cells collapsed to a single faster-migrating form, confirming that the mobility shift was due to increased phosphorylation (Figure 3B).

To identify the protein domain required for the nuclear localization of SRPK2, we created a set of deletion and point mutations in SRPK2. In particular to determine whether there was a link between translocation to the nucleus and phosphorylation of SRPK2, we generated point mutations in serine and tyrosine residues that were predicted phosphorylation targets according to the software *Scansite* [22]. The scheme of all the designed mutants is presented in Figure S3A. All the mutant proteins were examined for their intracellular localization both in untreated and in PQ-treated cells (Figure S3B). Interestingly and in contrast to SRPK1 [23], deletion of the spacer domain did not affect SRPK2 subcellular localization both in SH-SY5Y and in HeLa cells (Figure S3B and data not shown, respectively). As shown in Figure 3C, one mutant, SRPK2(S581A) was unable to relocalize to the nucleus upon PQ treatment. Quantification of the nucleus to cytoplasm (N/C) ratio for SRPK2(S581A) in PQ-treated cells indicated an approximately 3-fold reduced N/C ratio compared to wild type SRPK2 (Figure 3D). In contrast, the respective phosphomimetic mutation, S581D, was nuclear even in the absence of PQ. Serine 581 is located in the highly conserved C-terminal kinase domain within a putative casein kinase 2 consensus sequence (S/T-X-X-E/D, Figure 3E). Taken together, these results indicate that phopshorylation of serine 581 is required for nuclear accumulation of SRPK2.

### Nuclear SRPK2 modifies splice site selection

**Figure 4:**
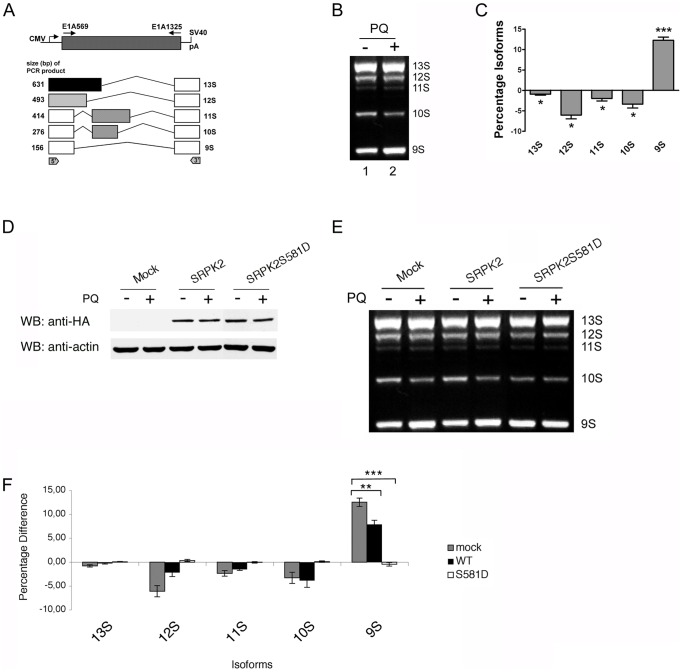
PQ treatment or nuclear SRPK2 affect splice site selection of the E1A minigene transcript. A. Schematic diagram illustrating the structure of the construct and the splicing pattern of the E1A minigene reporter. Arrows mark the position of the primers used for PCR analysis. The alternative 5′ splice site and splicing events that generate the different mRNA variants are indicated. B. Representative agarose gel of the splicing assay. SH-SY5Y cells were transiently transfected with the E1A reporter plasmid. 24 h after transfection cells were treated PQ. Total RNA was then isolated and the alternative splicing pattern of the E1A transcripts was determined by RT-PCR. The treatment with PQ induced an increase of the production of the 9 S transcript variant with respect to the other isoforms. C. Densitometric analysis of the E1A splicing products (mean ± S.E., n = 3) in cells treated with vehicle or with PQ. *** indicates p<0.001, and * indicates D. p<0.05, compared with control by paired, two-tailed Student's t test. E. Western blot analysis of the expression level of HA-tagged wild type and the S581D mutant in untreated and in PQ-treated SH-SY5Y cells. Blots were probed with an anti-HA antibody. Actin was used as loading control. F. SH-SY5Y cells were transiently transfected with the E1A splicing reporter minigene alone or together with expression plasmids coding for HA-tagged wild type SRPK2 or with the S581D mutant. RNA and protein fractions were simultaneously prepared. The alternative splicing pattern of the E1A transcripts was determined by RT-PCR. G. Densitometric analysis of the splicing products (mean ± S.E., n = 3) in untreated and PQ-treated cells. The relative levels of 13 S, 12 S, and 9 S mRNAs were quantitated as described in Materials and Methods. *** indicates p<0.001, * * indicates p<0.01, by one-way ANOVA and Dunnett's post test.

Since PQ treatment leads to the translocation of SRPK2 from the cytoplasm to the nucleus, and to hyperphosphorylation and accumulation of SR proteins in nuclear speckles, we reasoned that these events may affect splice site selection by modifying the balance between SR proteins and other splicing regulatory proteins, e.g. hnRNP proteins. To test this hypothesis we used the Adenovirus 2 E1A minigene whose pre-mRNA can be processed into five well-characterized mRNAs (Figure 4A). Three major forms, 13 S, 12 S and 9 S derive from the selection of alternative 5′ splice sites [24]. Two minor forms, 11 S and 10 S, involve the minor usage of a cryptic upstream 3′ splice site localized in an intron [25]. The splice site selection on this pre-mRNA depends on the relative concentrations of hnRNP A1 and SR proteins [12]. As illustrated in Figure 4B (lane 1), transfection of the E1A minigene in SH-SY5Y cells generated variable amounts of the five reported RNA species, reflecting the differential usage of the alternative 5′ and 3′ splice sites. PQ treatment resulted in a shift in splicing favoring use of the most distal 5′ splice site that gives raise to the 9 S RNA isoform (Figure 4B, lane 2, and 4C).

Next we asked whether the modification of the alternative splicing pattern of the E1A minigene observed in PQ-treated cells was a consequence of the increased level of SRPK2 in the nucleus. To this end, cells were co-transfected with the E1A minigene and with constructs expressing HA-tagged wild type HA–SRPK2 or the mutant HA–SRPK2(S581D) (Figure 4D). The splicing pattern of E1A was then compared between untreated cells and cells treated with PQ. As shown in Figure 4E and 4F, expression of the phospho-mimetic mutant SRPK2(S581D) in untreated cells increased production of the 9 S splice variant. Interestingly, when expressing the nucleus-enriched SRPK2(S581D) mutant, treatment with PQ did not further increase the production of the 9 S isoform indicating that the nuclear localization of SRPK2 is sufficient to induce changes in the splicing pattern of the E1A transcript.

### DNA damage induces nuclear translocation of SRPK2 and changes in splice-site selection

**Figure 5:**
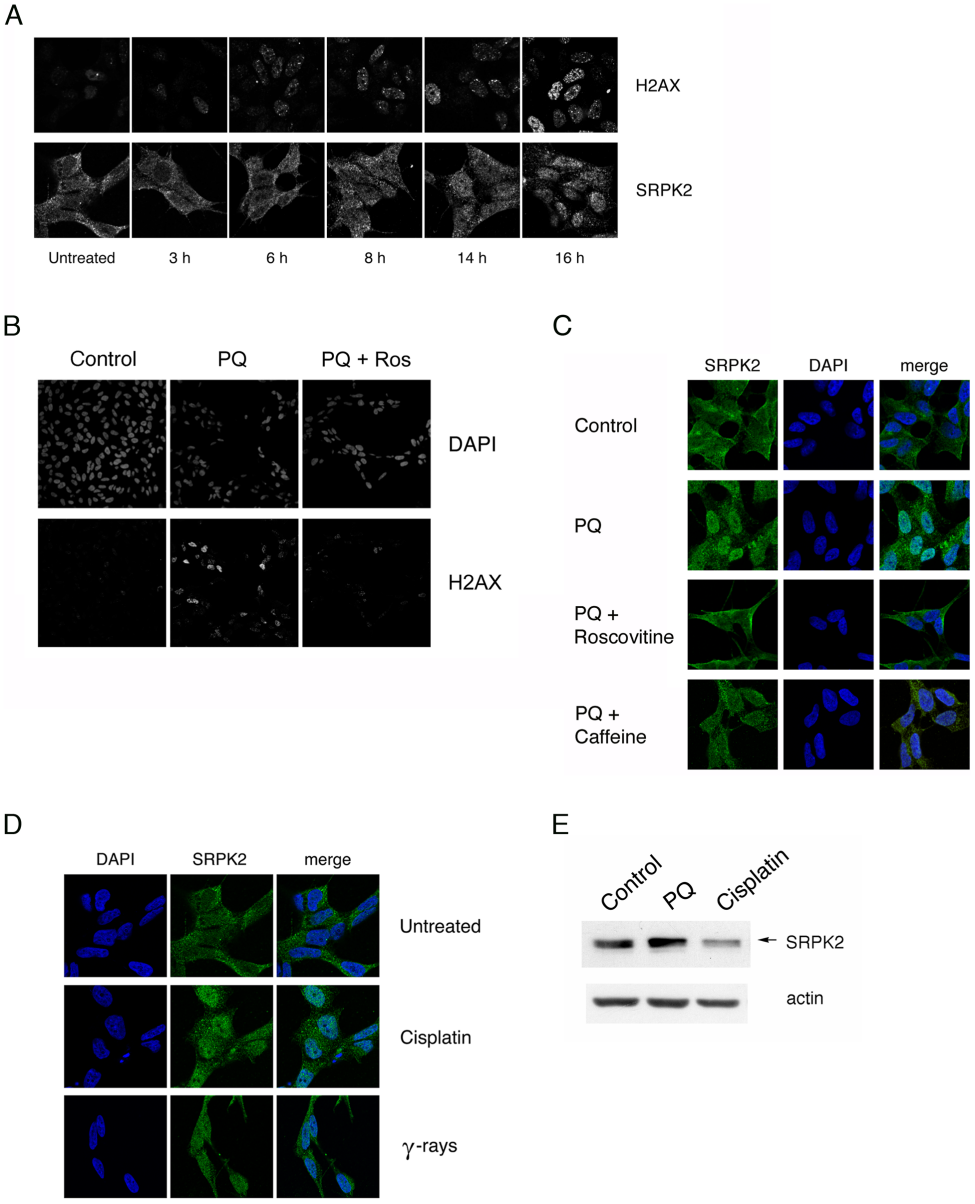
Genotoxic stress induces nuclear accumulation and phosphorylation of SRPK2. A. PQ induces DNA damage. Representative confocal micrographs of SH-SY5Y cells treated with PQ and fixed at the indicated time-points. Cells were stained with an anti-γH2AX antibody (*upper row*) or with an antibody specific for SRPK2 (*lower row*). B. Inhibition of PQ-induced H2AX phosphorylation by roscovitine. Representative confocal micrographs of SH-SY5Y cells incubated with 10 µM roscovitine and PQ treatment. ãH2AX was detected by immunocytochemistry; nuclei were stained with DAPI. C. Inhibition of the DDR blocks nuclear localization of SRPK2. Representative confocal micrographs of control SH-SY5Y cells (*first row*), or SH-SY5Y cells incubated with PQ alone (*second row*), or with PQ and with 10 µM roscovitine (*third row*), or with PQ and 10 mM caffeine (*forth row*). SRPK2 was detected by immunocytochemistry; nuclei were stained with DAPI. D. Genotoxic treatments induce nuclear localization of SRPK2. Representative confocal micrographs of untreated SH-SY5Y cells (*upper row*) or cells treated with 20 µM cisplatin for 18 h (*middle row*) or irradiated with 10 Gy (*lower row*) were stained with DAPI and with an anti-SRPK2 antibody. E. Genotoxic treatments induce hyperphosphorylation of SRPK2. Western blots of total cell extract prepared from untreated SH-SY5Y cells or from cells treated with PQ, or with cisplatin for the indicated times. The blot was probed with an anti-SRPK2 antibody. Actin was used as loading control. The slower migrating band is indicated by an arrow.

PQ is an uncoupler of the mitochondrial electron transport chain that induces superoxide formation [1] and oxidative stress [26]. Consequences of oxidative stress are DNA lesions, including oxidized DNA bases, abasic sites, and single-strand and double-strand breaks (SSBs and DSBs). Therefore, we wondered whether the translocation of SRPK2 to the nucleus might be a consequence of the cellular response to DNA damage. Indeed, the activation of the DNA damage response (DDR) is suggested by the observation that several genes related to this pathway are differentially expressed in PQ-treated cells (Table 1). To monitor PQ-induced DNA damage, we checked the phosphorylation status of the Ser-139 residue of the histone variant H2AX, forming ãH2AX (for review see [27]) in cells treated with PQ for different lengths of times (Figure 5A). The time of appearance of ãH2AX foci in PQ-treated cells paralleled the relocalization of SRPK2 from the cytoplasm to the nucleus, suggesting that the two events may be connected. To further strengthen the link between intracellular distribution of SRPK2 and the response to DNA damage, we tested the effect of chemical inhibitors of some of the signaling kinases involved in this pathway. Recent studies in yeast and metazoa have revealed that cyclin-dependent kinases (CDKs) play an active role in the response to DNA damage [28], [29]. We thus treated SH-SY5Y cells with the general CDK inhibitor roscovitine. Consistent with earlier reports [30], [31], incubation with roscovitine strongly reduced the appearance of γH2AX foci after PQ treatment (Figure 5B). Interestingly, in the presence of roscovitine, PQ treatment no longer promoted the accumulation of SRPK2 in the nucleus (Figure 5C). A similar effect was obtained in the presence of caffeine, a potent inhibitor of ATM and ATR, crucial signaling kinases that mediate the response to DNA damage (Figure 5C). Next, we verified whether other genotoxic treatments also promoted nuclear translocation of SRPK2. As shown in Figure 5D, treatment with 20 µM cisplatin for 18 hours or irradiation with 10 Gy induced both accumulation of endogenous SRPK2 in the nucleus and its hyperphosphorylation (Figure 5E). Similar results were obtained in HEK 293 cells (data not shown). Taken together these results show that activation of the DDR results in an increase of the nuclear pool of SRPK2.

**Table 1:** Differentially expressed genes involved in DNA damage response.

Accession Number	Gene Symbol	Description	Log2Fold expression	P.Value
NM_032043	BRIP1	BRCA1 interacting protein C-terminal helicase 1	−1.00	3,53×10^−3^
NM_005194	CEBPB	CCAAT/enhancer binding protein (C/EBP), beta	1.80	4,47×10^−8^
NM_001806	CEBPG	CCAAT/enhancer binding protein (C/EBP), gamma	1.03	5,85×10^−9^
NM_022111	CLSPN	claspin homolog (Xenopus laevis)	−1.14	1,55×10^−3^
NM_000107	DDB2	damage-specific DNA binding protein 2, 48 kDa	1.24	2,09×10^−9^
NM_004083	DDIT3	DNA-damage-inducible transcript 3	3.46	8,47×10^−8^
NM_019058	DDIT4	DNA-damage-inducible transcript 4	2.51	3,46×10^−7^
NM_001031716	OBFC2A	oligonucleotide/oligosaccharide-binding fold containing 2A	1.19	1,14×10^−4^
NM_006502	POLH	polymerase (DNA directed), eta	1.45	1,02×10^−9^
NM_018137	PRMT6	protein arginine methyltransferase 6	−1.14	5,59×10^−9^
NM_004219	PTTG1	pituitary tumor-transforming 1	−1.03	1,19×10^3^
NM_031271	TEX15	testis expressed 15	−1.59	1,15×10^3^

**Figure 6:**
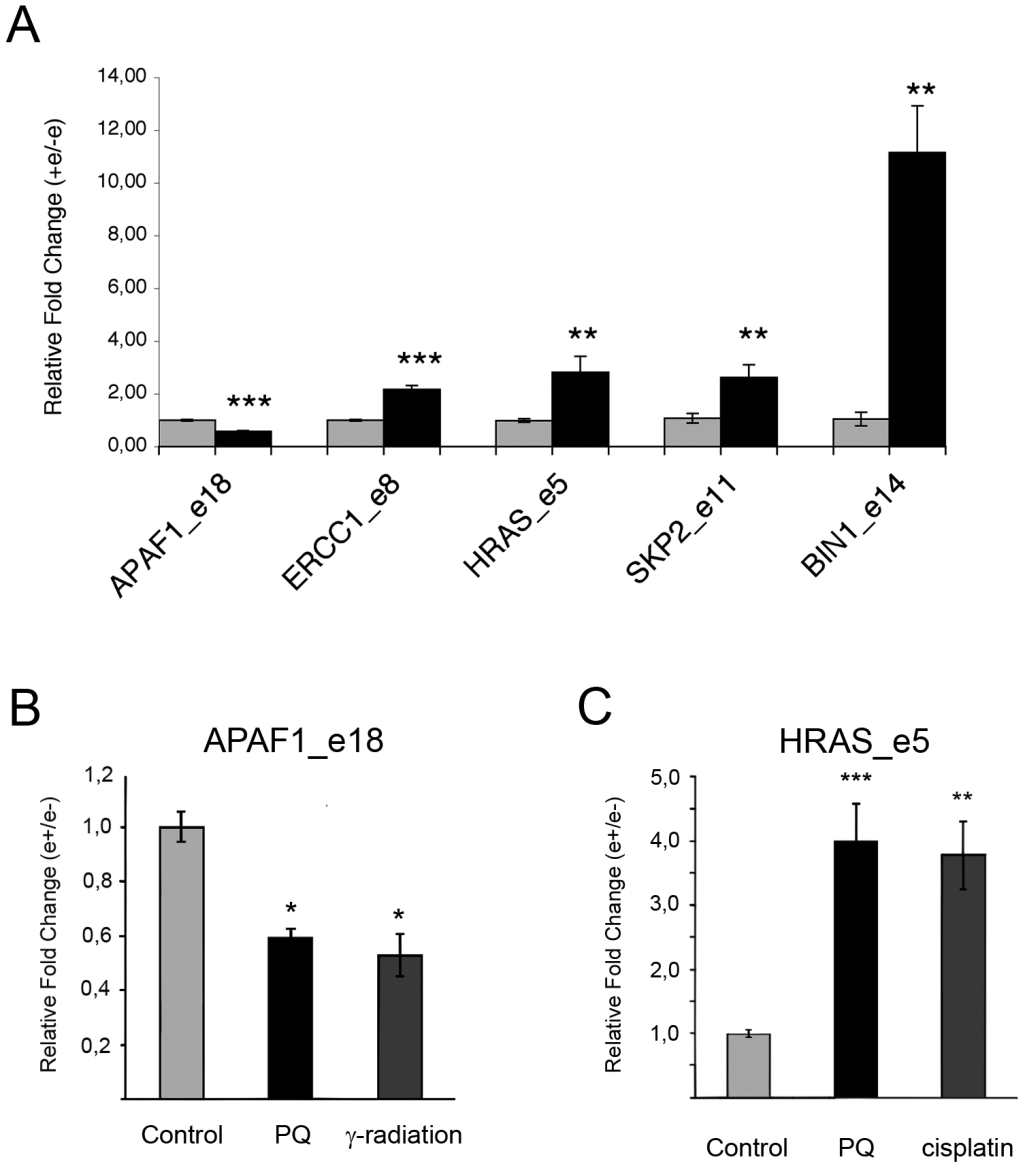
Genotoxic stress modifies alternative splicing of endogenous genes. A. SH-SY5Y cells were incubated with vehicle or with PQ as described in Material and Methods. The bar graph represents the quantification of the RT-PCR splicing analysis of the alternatively spliced regions of the APAF1 (exon 18, e18), H-RAS (exon 5, e 5), ERCC1 (exon 8, e 8), SKP2 (exon 11, e 11), and BIN1 (exon 14, e14) transcripts. The indicated splice forms were subcloned and sequenced to verify their identity. The inclusion or the skipping of variable exons after PQ treatment (black bars) was normalized relative to that observed in the respective controls (light grey bars). Error bars indicate the standard error three biological replicates. The asterisks represents the result of two-tailed t-test: *** indicates p<0.001, ** indicates p<0.01. B. RT-PCR splicing analysis of the alternatively spliced exon 18 (e18) of the APAF1 transcript in SH-SY5Y cells treated with vehicle (control), PQ, or γ-radiation. The asterisks represents the result of one-way ANOVA and Tukey's post test: * indicates p<0.05. C. RT-PCR splicing analysis of the alternatively spliced exon 5 (e5) of the H-RAS transcript in SH-SY5Y cells treated with vehicle, PQ, or cisplatin as described in Material and Methods. The asterisks represents the result of one-way ANOVA and Dunnett's post test: *** indicates p<0.001, ** indicates p<0.01.

The DDR includes gene expression programs that control cell cycle, DNA repair and apoptosis. Alternative splicing is known to regulate many key genes in these pathways [3], [32]. Consistent with this notion, several putative alternative splicing events that were identified in our splicing-sensitive microarray analysis of PQ-treated cells occurred in genes involved in DNA repair, cell cycle, and cell death [16]. To experimentally validate a subset of these splicing events affecting internal exons, we peformend RT-PCR analysis. As illustrated in Figure 6A, PQ treatment induced changes in the AS pattern of genes involved in apoptosis (APAF1, BIN1), cell cycle control (H-RAS; SKP2), and DNA repair (ERRC1). Interestingly, in the case of APAF1 and HRAS the ASE responded not only to PQ but also to γ-radiation or cisplatin (Figure 6B and 6C, respectively). Moreover similarly to PQ, cisplatin treatment of SH-SY5Y transiently transfected with the E1A splicing reporter construct stimulated the use of the most distal 5′ splice site, which gives rise to the 9 S isoform. In conclusion, these experiments strongly suggest that DNA damage can trigger changes in the splicing pattern of cellular genes by modifying the ratio between cytoplasmic and nuclear SRPK2.

## Discussion

In this report we investigated the molecular mechanism underlying the changes in alternative splicing that are induced by PQ treatment of SH-SY5Y neuroblastoma cells. We show that PQ leads to the phosphorylation and the accumulation of SRPK2 in the cell nucleus, and to increased phosphorylation of SR proteins. Relocalization of SRPK2 correlates with changes in the alternative splicing pattern of the E1A splicing reporter and of endogenous transcripts.

The molecular mechanisms that allow various stress stimuli to be transmitted to the nucleus are still only partially understood. Most research has concentrated on the elucidation of signal transduction pathways that target transcription factors. A few reports have however indicated that alternative pre-mRNA splicing is also a target of stress signaling. Osmotic stress was shown to induce the relocalization of hnRNPA/B the cytoplasm, resulting in changes in the alternative splicing pattern of an adenovirus E1A pre-mRNA splicing reporter [12]. Osmotic stress also promotes nuclear accumulation of SRPK1, the ubiquitously expressed paralogue of SRPK2 [15].

We demonstrate here that a phosphomimetic substitution of a serine residue located in the C-terminal kinase domain is necessary and sufficient to promote the accumulation of SRPK2 in the nucleus in the absence of PQ. In contrast, substitution of this serine with a non-phosphorylatable alanine residue prevents PQ-induced translocation. Interestingly, we found that caffeine and roscovitine prevent nuclear accumulation of SRPK2 upon PQ treatment. Moreover, we observed the formation of γH2AX foci in PQ-treated cells. The kinetics of foci appearance closely correlated with the nuclear accumulation of SRPK2, suggesting that SRPK2 relocalization may be due to the activation of the DDR. Consistent with this idea, we found that cisplatin treatment and γ irradiation also induced an increase in nuclear SRPK2.

**Figure 7:**
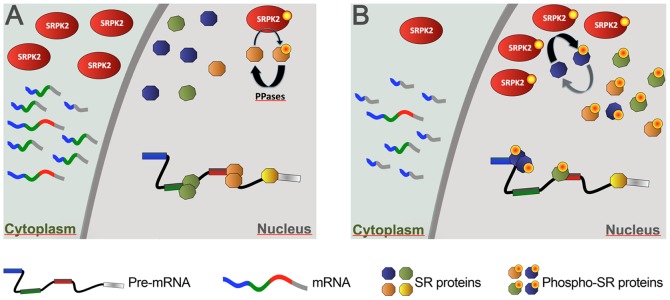
Model of SRPK2 action inthe modulation of stress-dependent splicing. A. Under normal conditions SRPK2 is mainly located in the cytoplasm. Its low level in the nucleus is counteracted by phosphatases that keep SR proteins in a hypophosphorylated state. B. Upon activation of stress-dependent signalling (f. ex. induced by DNA damage) SRPK2 is phosphorlyated in serine 581 and translocates to the nucleus. Here, the increased SRPK2 concentration overrides dephosphorlyation by PPases and leads to hyperphosphorylation of SR proteins. Since hyperphosphorylation of the RS domain is detrimental for splicing activity, this results in the reduction of the active pool of SR proteins and to a change in the balance between SR proteins and hnRNP proteins thus modifying the choice of alternative splice sites.

The biological function of SR proteins is regulated by cycles of reversible phosphorylation [33]. Not surprisingly, phosphorylation of SR protein is under the tight control of specific protein kinases and phosphatases [34]. Increased amounts of SRPK2 in the nucleus may alter the balance between SR protein kinases and phosphatases leading to the hyperphosphorylation of SR proteins, which in turn modulates splice site selection (Figure 7). Consistent with this idea, we observed increased phosphorylation of most SR proteins and a shift in splice site selection of the E1A minigene reporter towards the 9 S mRNA variant. Similar effects on SRPK1 localization and E1A splicing are induced by osmotic stress [15]. Therefore, the redistribution of SRPKs may represent a more general mechanism by which cells transduce stress signals to change alternative splicing of key genes in cell fate determination.

## Materials and Methods

### Constructs

pME-HAmSRPK2 [45] was used as a template to derive all the SRPK2 mutants. The spacer-deleted mutant was obtained by double digestion of pME-HAmSRPK2 with *Bam*HI and *Hind*III followed by fill-in and ligation of the vector. The N-terminus deleted was obtained by a first amplification with the oligonucleotides Nterm-FW (5′- GTCGACGCGGCCGCGGAGATTCTGGGGTCAGATG -3′) and *Hind*III-REV (5′- CGGAAGCTTCTGCAGAGGT -3′)), and then subcloning in pME-HAmSRPK2 digested with *Hind*III*/Sal*I. All other mutants were obtained thorough site directed mutagenesis (for the complete list of oligonucleotide sequences used in the mutagenesis see supplementary table S2). The plasmid p-hSRPK2-FLAG was obtained by inserting a FLAG tag in pCMV6-XL5-hSRPK2 (SC107542, OriGene). PCR was performed using the oligonucleotides hSRPK2-fw (5′-TATGTCAGTTAACTCTGAGAAGTCGT-3′) and hSRPK2-rev (5′-ACTACTTATCGTCGTCATCCTTGTAATCAATGTTAACAGAATTCAACCAAGGAT-3′), and the Phusion Hot Start High-Fidelity DNA Polymerase (F-540 S, Finnzymes). The PCR product was cleaved with NotI and inserted in pCDNA3. All the constructs were verified by nucleotide sequencing.

To generate silencing constructs for SRPK1 and SRPK2, sequences coding for short hairpin RNAs (shRNAs) were inserted as double-stranded oligonucleotides into pSUPuro between the *Bgl*II and *Hind*III sites as described [35], [36]. In each construct, the sense and antisense sequences of the target sequence (SRPK1: 5′- GGACAAAGCCCAAAGGAAA-3′; SRPK2: 5′- GCGAGAAGCTGAAAGGAAA-3′) are separated by a 9 nt spacer (TTCAAGAGA) that allows the formation of a hairpin loop. These vectors are referred to as pSUPuro-SRPK1 and pSUPuro-SRPK2. To generate the pLV-TH SRPK1 and SRPK2 plasmids, the pSUPuro plasmids were digested with *Bst*XI and *Sal*I and the H1-shRNA cassette was inserted into the same sites of pLV-TH [37].

### Cell culture and drug treatments

Human neuroblastoma SH-SY5Y were cultured in D-MEM/F-12 medium with GlutaMAX™ (Gibco, Invitrogen, UK), 10% FBS, 100 Units/mL penicillin G, 100 µg/ml streptomycin/penicillin (Euroclone, Milano, Italy). After having reached confluence, cells were reseeded at 3×10^6^cells in 100 mm dish. Paraquat (N,N′-dimethyl-4,4′-bipyridinium dichloride, Sigma-Aldrich) treatment was carried out essentially as described in (Maracchioni, et al., 2007) but for 18 h at 0.75 mM concentration. Treatment with cisplatin (Sigma-Aldrich) was performed for 18 h at 20 µM concentration. Roscovitine was used at a concentration of 10 µM and caffeine at 10 mM.

Transfections for RNA extraction or immunoblotting were performed with polyethylenimine (PEI, 40,872-7, Sigma, 100 mM in H_2_0 pH 7.00) according to the manufacturer's instruction. The transfected cells were incubated for further 24 or 48 h before lysis. For immunofluorescence analysis, cells were seeded in a 6-well plate containing a coverslip in each well. The next day 3 µg plasmid DNA was transfected using Transfast (Promega).

### RNA interference

LV-ttR Krab-dsRed-transduced SH-SY5Y cells were incubated with recombinant lentiviruses containing the shRNA expression cassettes for SRPK1 and SRPK2 [37]. To allow fluorescence-activated cell sorting of dsRed/GFP double positive cells, doxycyclin was added to the growth medium 48 h before sorting. Cell sorting positive cells was performed on a FACS ARIA apparatus (Becton Dickinson, Franklin Lakes, NJ, USA). To generate the inducible SRPK1/2 double knockdown cell line, the sorted SH-SY5Y SRPK2 shRNA cell line was retransduced with LV-TH SRPK1 shRNA viral supernatant. To induce silencing of SRPKs cells were incubated for five days with 5 µg/ml doxycyclin.

### Immunofluorescence and confocal microscopy analysis

Cells grown on the glass coverslips were fixed with 4% paraformaldehyde in PBS for 10 min and permeabilized with CKS solution (Hepes 20 mM, sucrose 300 mM, NaCl 50 mM, MgCl2 3 mM, Triton 0,2%) cold for 5 min. After blocking with FBS 10% in PBS with 0,05% Tween for 30 min, coverslips were incubated for 1 h in a humid chamber with the following primary antibodies in PBS containing 0,2% BSA: goat polyclonal anti-SRPK2 (P-19, sc-11308, Santa Cruz Biotechnologies), mouse monoclonal anti-SRPK2 (611118, BD Biosciences), mouse monoclonal anti-HA (Clone 6E2, 2367, Cell Signaling Technology), rabbit polyclonal anti-HA (Y-11, sc-805, Santa Cruz Biotechnologies), or mouse monoclonal anti-SC35 (S4045, Ascites Fluid, Sigma). After washing three times with PBS plus 0.2% BSA, the coverslips were stained with the respective fluor-conjugated secondary antibody in PBS and 0.2% BSA. The secondary antibodies were: Alexa 488-conjugated donkey anti-goat (A11055, Molecular Probes), Alexa 488-conjugated goat anti-mouse (A11001, Molecular Probes), Alexa 488-conjugated goat anti-rabbit IgG (A-11070, Molecular Probes), and Alexa 546-conjugated donkey anti-mouse (A10036, Molecular Probes). After dark incubation for 1 h, coverslips were washed three times with PBS plus 0.2% BSA, incubated in a solution containing 4,6-diamidino-2-phenylindole (DAPI, D9542, Sigma) 1 µg/mL in PBS for 10 min at RT, and mounted with FluorSave Reagent (345789, 20 mL, Calbiochem). The fluorescence 8-bit images were collected with a Leica TCS SP2 AOBS confocal microscope with the 63× oil immersion objective. Quantification of the data was done using LSC software: 12-bit images were acquired by using the same setting parameters for all the samples (gain, offset); for each field, five different xy sections along the z axis were acquired. Measurements were obtained for the nuclear fluorescence (Sn), the total cell fluorescence (Sc), the area of the nucleus (An), and area of the cell (Ac). The cytoplasmic (C′) amount of signal was calculated as: C′ = 1 - [(An Sn)/(Ac Sc)]. C/N ratios were calculated as C/N = C′/(1–C′).

### Western blot analysis

Cells were lysed in lysis buffer (Tris HCl 50 mM pH 6.80, NaCl 150 mM, 1% NP40, 5 mM EGTA, 5 mM EDTA) with protease inhibitors (Complete PIC, 04 693 116 001, Roche) and phosphatase inhibitors (PhIC-1, P2850, Sigma; PhIC-2 P5726, Sigma). Phosphatase treatment of total protein lysates was performed with calf intestinal alkaline phosphatase was used (CIP, New England Biolabs) following manufacturer's instructions.

Proteins were separated in SDS-polyacrylamide gels and transferred to nitrocellulose membranes (Whatman GmbH). Membranes were blocked using 5% non fat dried milk in PBST (0.1% (v/v) Tween 20 in 1× PBS) for 1 h at room temperature and incubated with a primary antibody. The following primary antibodies were used: mouse monoclonal anti-CPSF73K [38], mouse monoclonal antibody mab 104 [16] mouse monoclonal anti-SR proteins (clone 16H3, Invitrogen), mouse monoclonal anti-SRp20 (clone 7B4, Invitrogen), mouse monoclonal anti-ASF/SF2 (clone 96, Invitrogen), mouse monoclonal anti-ß actin (ab8226, Abcam), mouse monoclonal anti-SRPK2 (BD Biosciences), mouse monoclonal anti-hnRNP K/J (clone 3C3, Sigma), mouse monoclonal anti-hnRNP A1 (clone 4B10, Abcam), rabbit polyclonal anti-hnRNP H (Novus Biological), mouse monoclonal anti-hRNP C1/C2 (clone 4F4, Sigma), goat polyclonal anti-LDH (Chemicon International), mouse monoclonal anti-FLAG-M2 (Sigma) mouse monoclonal anti-HA (Clone 6E2, Cell Signaling Technology). After washing membranes were incubated with peroxidase-conjugated secondary antibody anti-mouse IgG (GE Healthcare), anti-goat IgG (Pierce), anti-rabbit IgG (Pierce,), anti-mouse IgM (Santa Cruz Biotechnologies) and then detected with ECL reagents (GE Healthcare).

### RNA extraction and RT-PCR analysis of alternative splicing

All the splicing analyses were performed on three independent RNA preparations. Total RNA was from cultured cells extracted using TRIzol® Reagent (Invitrogen), and subsequently purified using silica membrane spin columns from RNeasy Mini kit (Qiagen). RNA quantity and purity were assessed using a NanoDrop® instrument (Thermo Fisher Scientific Inc.). 2 µg of total RNA were reverse-transcribed using MultiScriveTM Reverse Transcriptase (Applied Biosystems), random hexamers (Applied Biosystems), RNAsin Plus reagent (Promega) and dNTPs for 2 h at 37°C according to manufacture's instruction. PCR assay conditions were optimized for each gene with respect to primer annealing temperatures, primer concentration, and MgCl2 concentrations. The number of amplification cycles used for each reaction was determined to ensure that transcript amplification was within a linear range (25 to 35 cycles). Gene specific primer sequences are listed in Supporting Information, Table S1. Quantification of the PCR products was performed with a 2100 Bioanalyzer (Agilent Technologies). Statistical analysis was performed using GraphPad Instat software (GraphPad Software Inc.). The amplified PCR products were cloned in pGEM T Easy Vector System (Promega, Madison, USA) and sequenced by BMR Genomics.

### E1A splicing reporter assays

SH-SY5Y cells were either transfected with 5 µg of the reporter construct alone or cotransfected with 5 µg of pME-HA-SRPK2 constructs as described above. Bluescript vector was used to equalize the amount of total DNA transfected into the cell. Total RNA was extracted using Trizol (Invitrogen) and passed into RNeasy Mini Kit columns (Qiagen). After DNase treatment (Promega), cDNA was synthesized from 1 µg of total RNA in a 20 µl reaction with oligo-dT primer (Promega) and M-MLV Reverse Transcriptase (Promega). One µl was then used for PCR amplification with GoTaq Flexi DNA Polymerase (Promega) using the following E1A primers (5′-TGAGTGCCAGCGAGTAGAGTTTTCT-3′) and (5′-TCTGGCTCGGGCTCAGGCTCAGGTT-3′). Quantification of the PCR products was performed with a 2100 Bioanalyzer (Agilent Technologies). Statistical analysis was performed using GraphPad Instat software (GraphPad Software Inc.).

### Statistical analysis

Continuous variables are expressed as means ± SEM. The averages and SEMs were calculated from at least three independent experiments. Means of two-groups experiments were compared with t-test (unpaired or paired, according to the fact that data were respectively coupled or not). Means of multiple-groups experiments were compared using one-way ANOVA, Tukey post-test analysis, or Dunnett's post hoc test. All the analyses were performed using GraphPad prism 5.0 and GraphPad Instat 3. A P value of *P<0.05 was considered statistically significant, **P<0.01 was considered very significant, ***P<0.001 was considered extremely significant.

## Supporting Information

Figure S1 **Quantitative analysis of nuclear speckles in PQ-treated cells.** A. SH-SY5Y cells treated with vehicle or with PQ were immunostained with an antiSC35 monoclonal antibody. B. Anti-SC35 immunostained nuclear speckles in control SH-SY5Y cells (white bars) and in cells treated with PQ (black bars). While PQ affects neither the number nor the intensity of SC35-positive nuclear speckles, their size becomes significantly larger upon treatment. Quantification of the confocal micrographs was performed with the LSC Data Analysis Software on n = 5 representative cells. Samples measurements were obtained for the total area for each single speckle in each cell (pixel number in the considered region of interest ROI), mean amplitude of the pixel intensity for each single speckle in each cell (pixel intensity in the ROI), total number of speckles per each cell. Values shown are the mean ± SEM obtained from 17.6±3.3 nuclear speckles in five control cells and 14.0 ± 3.5 nuclear speckles in five PQ-treated cells respectively.
(TIFF)

Figure S2 **PQ treatment does not modify the expression level of hnRNP proteins.** Total extract of control or PQ-treated cells was probed with monoclonal antibodies to specific for hnRNP A1, hnRNP C1/C2, hnRNP K/J, or hnRNP H.
(TIFF)

Figure S3 **Intracellular distribution of SRPK2 mutant proteins.** A. Schematic diagram of the domain structure of wild type SRPK2 and of its mutant variants. B. Representative confocal micrographs of SH-SY5Y cell transfected with constructs expressing the indicated HA-tagged SRPK2 proteins.
(TIF)

Table S1 **Nucleotide sequences of the primers used for alternative splicing analysis.**
(DOC)

Table S2 **Oligonucleotides used to generate the SRPK2 mutants.**
(DOC)
