# Supplementary figures and images for: Paraquat Modulates Alternative Pre-mRNA Splicing by Modifying the Intracellular Distribution of SRPK2
Source: PLoS One. 2013 Apr 16;8(4):e61980. doi: 10.1371/journal.pone.0061980 (PMC3628584; doi:10.1371/journal.pone.0061980)

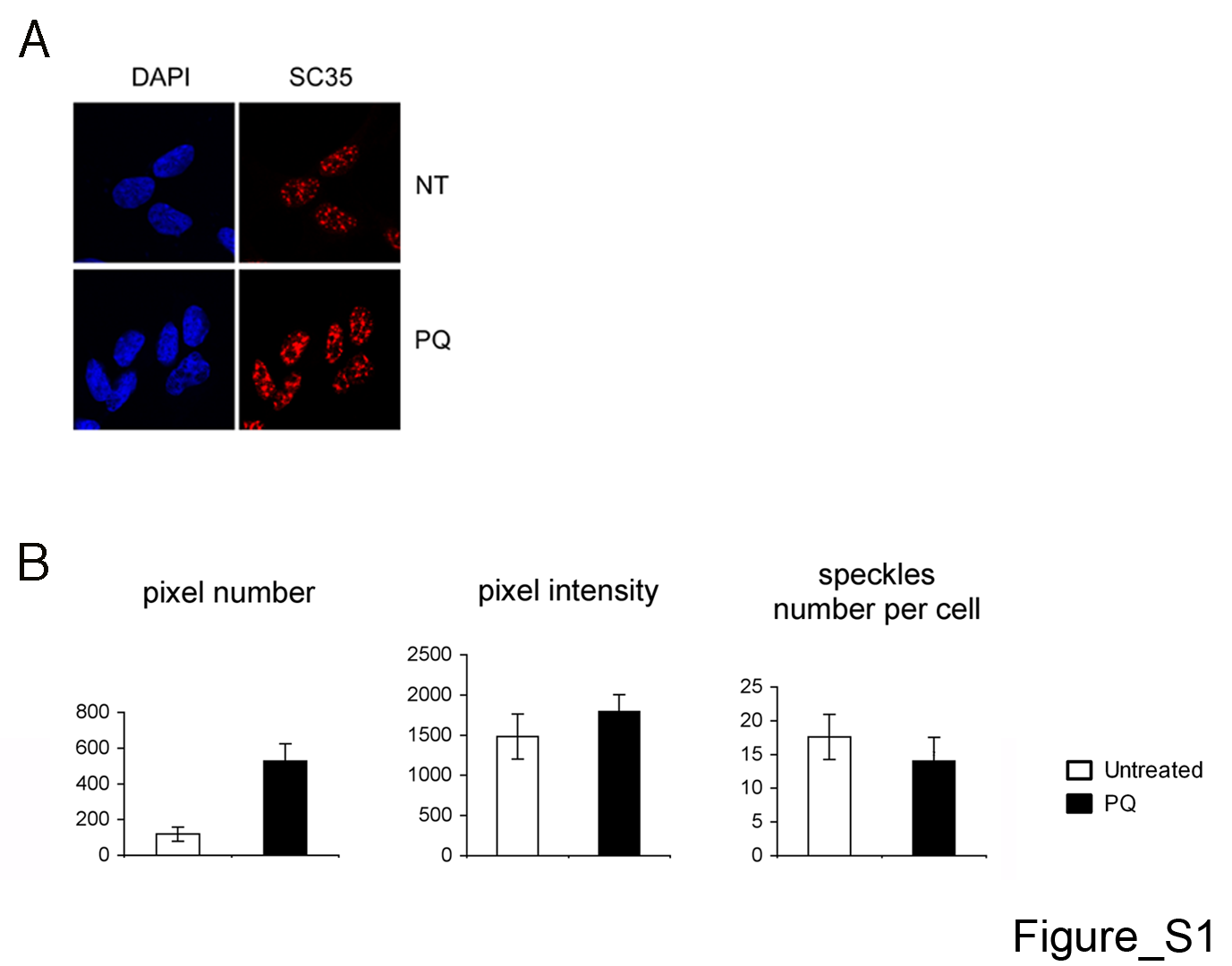

Supplement: Figure S1 — Quantitative analysis of nuclear speckles in PQ-treated cells. A. SH-SY5Y cells treated with vehicle or with PQ were immunostained with an antiSC35 monoclonal antibody. B. Anti-SC35 immunostained nuclear speckles in control SH-SY5Y cells (white bars) and in cells treated with PQ (black bars). While PQ affects neither the number nor the intensity of SC35-positive nuclear speckles, their size becomes significantly larger upon treatment. Quantification of the confocal micrographs was performed with the LSC Data Analysis Software on n = 5 representative cells. Samples measurements were obtained for the total area for each single speckle in each cell (pixel number in the considered region of interest ROI), mean amplitude of the pixel intensity for each single speckle in each cell (pixel intensity in the ROI), total number of speckles per each cell. Values shown are the mean ± SEM obtained from 17.6±3.3 nuclear speckles in five control cells and 14.0 ± 3.5 nuclear speckles in five PQ-treated cells respectively. (TIFF) [file pone.0061980.s001.tiff]

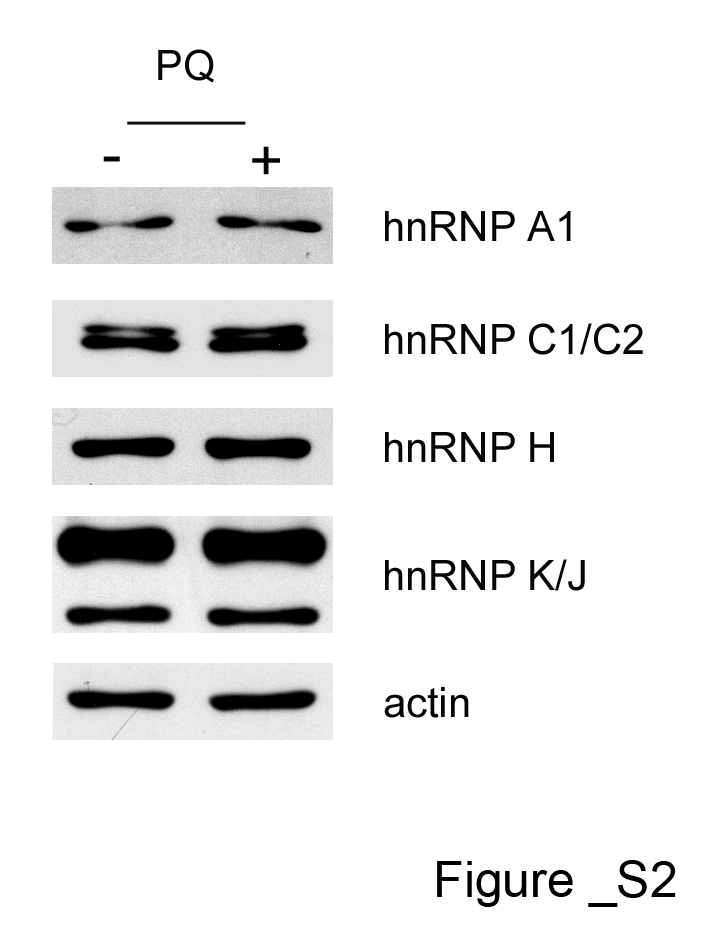

Supplement: Figure S2 — PQ treatment does not modify the expression level of hnRNP proteins. Total extract of control or PQ-treated cells was probed with monoclonal antibodies to specific for hnRNP A1, hnRNP C1/C2, hnRNP K/J, or hnRNP H. (TIFF) [file pone.0061980.s002.tiff]

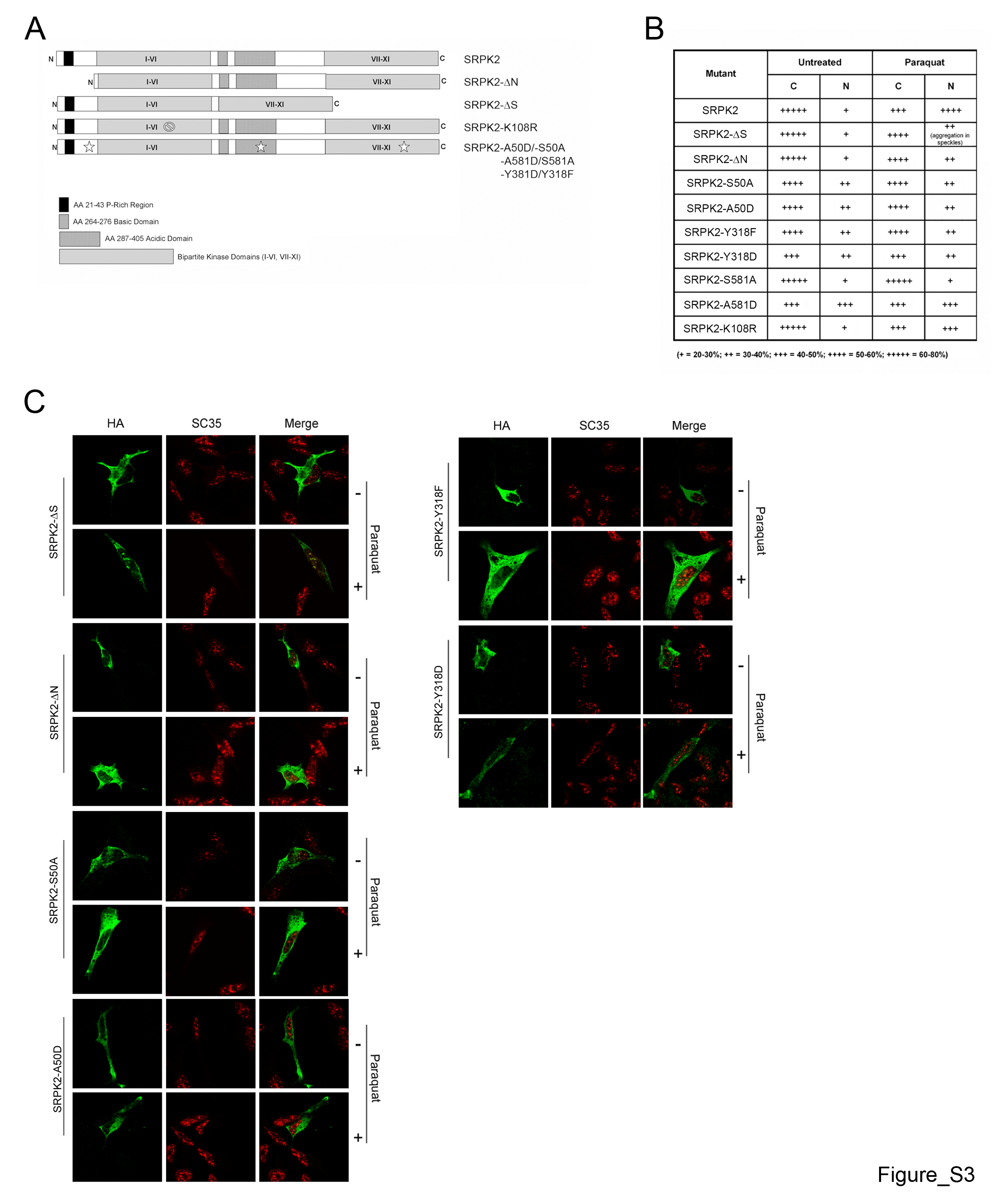

Supplement: Figure S3 — Intracellular distribution of SRPK2 mutant proteins. A. Schematic diagram of the domain structure of wild type SRPK2 and of its mutant variants. B. Representative confocal micrographs of SH-SY5Y cell transfected with constructs expressing the indicated HA-tagged SRPK2 proteins. (TIF) [file pone.0061980.s003.tif]
