# Supplementary material for: Paraquat Modulates Alternative Pre-mRNA Splicing by Modifying the Intracellular Distribution of SRPK2
Source: PLoS One. 2013 Apr 16;8(4):e61980. doi: 10.1371/journal.pone.0061980 (PMC3628584; doi:10.1371/journal.pone.0061980)
Supplement: Table S1 — Nucleotide sequences of the primers used for alternative splicing analysis. (DOC) [file pone.0061980.s004.doc]

Table_S1. Nucleotide sequences of the primers used for alternative splicing analysis .

| Gene ID | Primer | Sequence (5’-3’) | Exon anneling  (from Ensembl 62 release) |
| --- | --- | --- | --- |
| APAF1 | APAF1_Fw | CAGCTGATGGAACCTTAAAGC | ENSE00000818044 |
|  | APAF1_Rv | GTCTGGTCATCAGAAGATGTC | ENSE00000818050 |
| BIN1 | BIN1_Fw | CTGCAAAAGGGAACAAGAGC | ENSE00002377871 |
|  | BIN1_Rv | ATTCACAGTTGCTGGGAAGG | ENSE00002336898 |
| ERCC1 | ERCC1_Fw | CAACCTGCACCCAGACTACA | ENSE00000749054 |
|  | ERCC1_Rv | GGGCATAAGGCCAGATCTTC | ENSE00001419753  ENSE00000469632 |
| HRAS | HRAS_Fw | GTGGGGAACAAGTGTGACCT | ENSE00001206466 |
|  | HRAS_Rv | CATCAGGAGGGTTCAGCTTC | ENSE00001762405 |
| SKP2 | SKP2_Fw | CCAGCTCAACTACCTCCAACA | ENSE00000971195 |
|  | SKP2_Rv | GGCTGGACTTGAGTTTGGAA | ENSE00001862417 |
|  | SKP2 Rv | ATGAAGGCAAAGGGAAAACC | ENSE00001082238 |
