# Supplementary material for: Paraquat Modulates Alternative Pre-mRNA Splicing by Modifying the Intracellular Distribution of SRPK2
Source: PLoS One. 2013 Apr 16;8(4):e61980. doi: 10.1371/journal.pone.0061980 (PMC3628584; doi:10.1371/journal.pone.0061980)
Supplement: Table S2 — Oligonucleotides used to generate the SRPK2 mutants. (DOC) [file pone.0061980.s005.doc]

**Supplemental Table S2**

Oligoucleotides used to generate the SRPK2 mutants

| **Mutation** | **Primer Name** | **Primer sequence from 5’ to 3’**  **(Underlined is the degenerated nucleotide)** |
| --- | --- | --- |
| **K108R** | S50-FW | GTCGACGCGGCCGCGATGTCAGTTAACTCTGAGAAGT |
|  | HindIII-REV | CGGAAGCTTCTGCAGAGGT |
|  | MutK108R-FW | GATTTGTTGCAATGAGAGTTGTAAAAAGTGCC |
|  | MutK108R-REV | GGCACTTTTTACAACTCTCATTGCAACAAATC |
| **S50A** | S50-FW | GTCGACGCGGCCGCGATGTCAGTTAACTCTGAGAAGT |
|  | HindIII-REV | CGGAAGCTTCTGCAGAGGT |
|  | MutS50A-FW | GAGATTCTGGGGGGCAGATGATGAGGAGC |
|  | MutS50A-REV | GTCCCTCATCATCTGCCCCCAGAATCTC |
| **S581A** | SacI-FW | TGGCATTTGAGCTCGCCAC |
|  | SacII-REV | GGCCGCGGGAATTCGATTA |
|  | MutS581A-FW | GTTCGAACCGCATGCTGGGGAAGACTATTC |
|  | MutS581A-REV | GAATAGTCTTCCCCAGCATGCGGTTCGAAC |
| **Y318F** | Y318-FW | AGTTGCTGGAGAAACGCCTA |
|  | Y318-REV | ACAAGCATTTCCCAGGTCAG |
|  | MutY318F-FW | CAGGATGGAGAGTTCCAGCCGGAGGTGAC |
|  | MutY318F-REV | CACCTCCGGCTGGACTCTCCATCCTGCCTC |
| **Y318D** | Y318-FW | AGTTGCTGGAGAAACGCCTA |
|  | Y318-REV | ACAAGCATTTCCCAGGTCAG |
|  | MutY318D-FW | CAGGATGGAGAGTGCCAGCCGGAGGTGAC |
|  | MutY318D-REV | CACCTCCGGCTGGCCTCTCCATCCTGCTC |
| **A50D** | S50-FW | GTCGACGCGGCCGCGATGTCAGTTAACTCTGAGAAGT |
|  | HindIII-REV | CGGAAGCTTCTGCAGAGGT |
|  | MutA50D-FW | GAGATTCTGGGGGACGATGATGAGGAGCA |
|  | MutA50D-REV | GCTCCTCATCATCGTCCCCCAGAATCTC |
| **A581D** | S50-FW | GTCGACGCGGCCGCGATGTCAGTTAACTCTGAGAAGT |
|  | HindIII-REV | CGGAAGCTTCTGCAGAGGT |
|  | MutA581D-FW | GTTCGAACCGCATGATGGGGAAGACTATTC |
|  | MutA581D-REV | GAATAGTCTTCCCCATCATGCGGTTCGAAC |
